# Selva Zoque, Mexico: an important Mesoamerican tropical region for reptile species diversity and conservation

**DOI:** 10.3897/zookeys.1054.67916

**Published:** 2021-08-03

**Authors:** José Luis Aguilar-López, Ricardo Luría-Manzano, Eduardo Pineda, Luis Canseco-Márquez

**Affiliations:** 1 Red de Biología y Conservación de Vertebrados, Instituto de Ecología A. C., Carretera antigua a Coatepec 351, El Haya, Xalapa, C.P. 91073, Veracruz, México; 2 Departamento de Ecologia, Instituto de Biociências, Universidade de São Paulo, Rua do Matão, Travessa 14, Cidade Universitária, 05508-090, São Paulo, São Paulo, Brazil; 3 Departamento de Biología Evolutiva, Laboratorio de Herpetología, Facultad de Ciencias, Universidad Nacional Autónoma de México, A.P. 70-399, C.P. 04510, México City, México

**Keywords:** Compositional similarity, conservation value, Data Deficient, reptile fauna, species composition, species richness

## Abstract

The Selva Zoque region is characterized by a great variety of ecosystems for which there is little information about reptile species diversity and their conservation status. This study is the first assessment of the species richness, composition, and conservation status of reptiles of this region. Additionally, this information is compared with that of seven other tropical regions in northern Mesoamerica. In total, 141 native reptile species belonging to 81 genera and 29 families are recorded for the Selva Zoque region. Sixty species (42% of the total) recorded in Selva Zoque are in high-risk categories according to the Mexican Ministry of the Environment, the highest number for the Mexican regions of Mesoamerica. According to the IUCN, six species are in high-risk categories, seven species are in Data Deficient, and 23 (16%) have not been evaluated yet. According to the Environmental Vulnerability Scores approach, 28 species (20%) are in the high vulnerability category. The Selva Zoque species composition is most similar to Los Tuxtlas and Lacandona regions, and most dissimilar to Sian Ka´an Biosphere Reserve. The reptilian fauna of Selva Zoque has a distinctive composition, with the highest number (11 species) of endemic reptiles in the northern Mesoamerican, and species from two biogeographic provinces: the Gulf of Mexico and the Mexican Pacific Coast. These results indicate that the Selva Zoque is the most diverse region in native reptile species in northern Mesoamerica, highlighting it as extremely important for the conservation of the reptile fauna at local (southern Mexico) and regional levels (northern Mesoamerica).

## Introduction

The 25 biodiversity hotspots identified by Myers et al. (2000) share two characteristics: each one harbors endemic plant species representing at least 0.5% of the global total, and have lost ≥ 70% of their primary vegetation. Of these regions, Mesoamerica ranks fifteenth in relation to the latter characteristic (80% of primary vegetation lost; Myers et al. 2000), and ranks third in deforestation rate among the 13 hotspots for which information is available (Brooks et al. 2002). Despite this scenario, some areas still remain covered by primary vegetation (FAO 2011), highlighting the ongoing conservation value of this region.

One such region is the Selva Zoque, composed by the Uxpanapa-Chimalapas zone (UC) and El Ocote Biosphere Reserve, is located on the Isthmus of Tehuantepec in the states of Veracruz, Oaxaca, and Chiapas in southern Mexico. The Selva Zoque region is the second largest extension of well-conserved tropical forest in northern Mesoamerica, is a Pleistocene refuge with high number of endemic species (Pérez-Farrera et al. 2016), contains a large variety of vegetation types (Peterson et al. 2003) and has a broad range of elevation spanning 100 to 2300 m a.s.l. Additionally, the Selva Zoque region, together with the protected area La Sepultura Biosphere Reserve, in the state of Chiapas, make up the La Selva Zoque-La Sepultura Priority Conservation Area (Arriaga et al. 2000b). Currently, detailed knowledge of the vertebrate species diversity in the entire Selva Zoque, or a large portion of it, is available only for birds (Peterson et al. 2003), mammals (Lira-Torres et al. 2012), and amphibians (Aguilar-López et al. 2016a). For other groups such as reptiles, our knowledge of species richness, species composition, and conservation status is limited to El Ocote Biosphere Reserve, where the reptile diversity has been revised on several studies (Reynoso et al. 2011; Luna-Reyes et al. 2017; Muñoz-Alonso et al. 2017) and scarce in UC zone. Herpetological expeditions have been carried out in the UC zone since at least the middle of the last century (Taylor 1951; Duellman 1960; Lynch and Wake 1989) but have focused mainly on amphibians. Moreover, herpetofaunal collection has been limited to few localities, and extensive portions of the region remain with no information.

Worldwide, habitat modification represents the most common threat to terrestrial reptile species, with one in five species included in high-risk categories of extinction (Vulnerable, Endangered, or Critically Endangered) by the IUCN. A further one in five species is listed in the Data Deficient category, and four of ten have not been evaluated according to the criteria of the Red List (Uetz et al. 2018; IUCN 2021). In Mexico, it is estimated that 13% of reptile species are threatened and for another 16% there is insufficient information (in Data Deficient category) to evaluate its extinction risk level (IUCN 2021). On the other hand, about half of the species are included in the high-risk of extinction categories on the species list compiled by the Mexican Ministry of the Environment (NOM-059-SEMARNAT-2010). Given the variety of tropical forests that cover the Selva Zoque region, along with its geographic location and environmental heterogeneity (Wendt 1987; de Teresa 2000; SEMARNAT 2001), a high reptile diversity may inhabit in this region, with a significant portion of species under high risk of extinction.

Based on a comprehensive review of databases, scientific literature, and fieldwork, we provide the first assessment of species richness, species composition and distribution, and conservation status of the reptile fauna inhabiting the Selva Zoque region. Additionally, we compared this information with other tropical regions on the northern end of Mesoamerica.

## Materials and methods

### Study site

**Figure 1.**
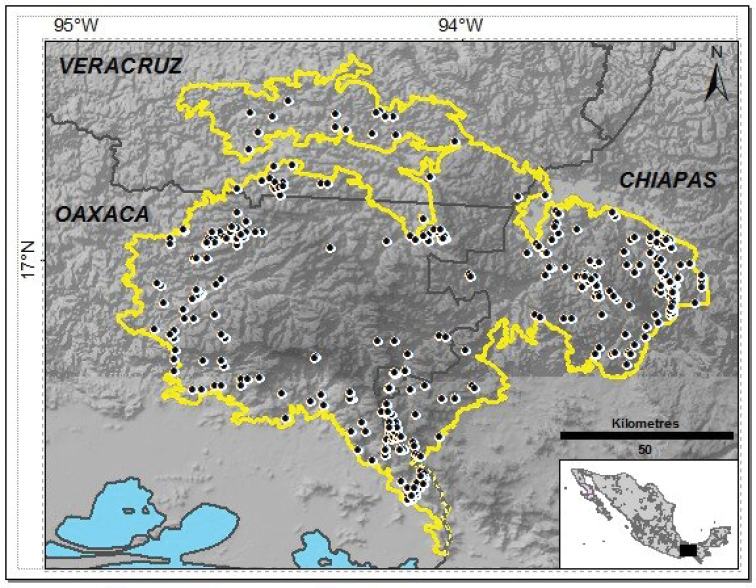
Location of the Selva Zoque region. The yellow line denotes our study area limits, the thin black line indicates state boundaries, and black circles denote localities in which reptiles have been recorded.

The Selva Zoque region is located in southern Mexico east of the Isthmus of Tehuantepec (Fig. 1). Mountains and hills dominate the region (Wendt 1987; SEMARNAT 2001; Ortiz-Pérez et al. 2004). The region is covered by several vegetation types, the main ones are evergreen tropical forest (100–1000 m a.s.l.), semi-evergreen tropical forest (600–1200 m a.s.l.), deciduous tropical forest (100–600 m a.s.l.), tropical montane cloud forest (1100–1800 m a.s.l.) and pine-oak forest (1800–2300 m a.s.l.) (Wendt 1987; SEMARNAT 2001). Mean annual temperature ranges from 12 to 23 °C and mean annual rainfall ranges from 800 to 4400 mm (Vidal-Zepeda 1990; SEMARNAT 2001). We delimited the study area using the polygon set by Arriaga et al. (2000a) for the Selva Zoque-La Sepultura Priority Conservation Area but excluded La Sepultura Biosphere Reserve (Fig. 1).

### Data collection

We consulted two databases between January 2017 and May 2021: the National Information System on Biodiversity (**SNIB**) curated by the National Commission for the Knowledge and Use of Biodiversity (**CONABIO**) and the Global Biodiversity Information Facility (GBIF; www.gbif.org). We also reviewed the scientific literature on reptiles from the study region (Navarro-Singüenza and Meave-Castillo 1998; Espinoza et al. 1999; Reynoso et al. 2011; Carmona-Torres 2013; Aguilar-López et al. 2014; Canseco-Márquez and Ramírez-González 2015; Scarpetta et al. 2015; Aguilar-López et al. 2016b; Gray et al. 2016; Luna-Reyes et al. 2017; Muñoz-Alonso et al. 2017; Campbell et al. 2018; del Moral-Flores et al. 2019). Additionally, we conducted fieldwork during 2013 and 2014 in surroundings of Arroyo Zarco, Uxpanapa, in Veracruz (17°11'N, 94°28'W), and San Francisco La Paz (17°5'N, 94°8'W), La Fortaleza (17°9'N, 94°13'W), and La Esmeralda (17°9'N, 94°46'W), in Santa María Chimalapa, Oaxaca. We surveyed areas with evergreen tropical forest and semi-evergreen tropical forest using standard visual encounter survey techniques (Crump and Scott 1994) during the day and at night. Cumulative sampling effort was 3250 person-hours.

We cross-checked records obtained from the three sources of information to avoid duplication, since the databases consulted might have records of the same specimens. Only records with precise geographic coordinates or detailed information about the collection and observation site were included, and from these records we compiled a general database.

To compare the data we compiled for the Selva Zoque region with the data from seven other tropical regions, we obtained information about reptile species richness and species composition from: 1) Los Tuxtlas in Veracruz (López-Luna 2017), 2) Las Choapas municipality in Veracruz (Aguilar-López and Canseco-Márquez 2006), 3) La Sepultura Biosphere Reserve in Chiapas (Nuñez-Orantes and Muñoz-Alonso 2000; Reynoso et al. 2011; Clause et al. 2020a, b), 4) the Lacandona rainforest in Chiapas (Hernández-Ordóñez et al. 2015), 5) the Calakmul Biosphere Reserve in Campeche (Calderón-Mandujano et al. 2010; Colston et al. 2015), 6) the Sian Ka´an Biosphere Reserve in Quintana Roo (Calderón-Mandujano et al. 2008), and 7) the Mayan Forest in Guatemala (Lee 1996; Campbell 1998).

### Data processing and analysis

To identify the spatial distribution of reptile records in the study region, we projected all geo-referenced records onto our study area polygon using ArcGIS software, version 10.0 (ESRI 2010). To determine the distribution and taxonomically standardize the data set of species that inhabit the Selva Zoque and the other tropical regions, we consulted the specialized literature documenting taxonomic changes and descriptions of new species (Wüster et al. 2005; Castoe et al. 2009; Köhler 2010; Linkem et al. 2011; Cadle and Savage 2012; Hedges and Conn 2012; Iverson et al. 2013; Porras et al. 2013; Köhler et al. 2014; Ruane et al. 2014; Blair et al. 2015; Meza-Lázaro and Nieto-Montes de Oca 2015; Card et al. 2016; Gray et al. 2016; Köhler et al. 2016; McCranie and Hedges 2016; Wallach 2016; Nieto-Montes de Oca et al. 2017; Campbell et al. 2018; Carbajal-Márquez et al. 2020; Jadin et al. 2020; McCranie et al. 2020; Reyes-Velasco et al. 2020; Ramírez-Reyes et al. 2021). Using the compiled data, we defined four distribution categories: species distributed outside Mesoamerica as widely distributed species (WD), species restricted to Mesoamerica (MA), species restricted to northern Mesoamerica (MAMx), and species restricted to one of the eight regions considered. We delimited Mesoamerica as suggested by Campbell (1999) and considered northern Mesoamerica as the zone corresponding to Mexico. To determine the extinction risk category for each species, we consulted the list of Species at Risk published by SEMARNAT, updated in 2018 (NOM-059-SEMARNAT-2010), the Red List maintained by the International Union for the Conservation of Nature (IUCN 2021), and the Environmental Vulnerability Score (EVS) proposed by Wilson et al. (2013). SEMARNAT’s categories are: Subject to Special Protection (Pr), Threatened (A), and Endangered (P). The IUCN’s three high-risk categories are: Vulnerable (VU), Endangered (EN) and Critically Endangered (CR); its low-risk categories are: Least Concern (LC) and Near Threatened (NT). We also included species in the Data Deficient (DD) category, and those Not evaluated (NE) by the IUCN. In addition, we consulted the EVS of Mexican reptile species that have been evaluated and assigned to one of three categories of vulnerability to environmental degradation: low (3–9), medium (10–13) and high (14–19). For the Mayan Forest in Guatemala, we were only able to assign the IUCN categories since the area lies outside of SEMARNAT’s jurisdiction and there is not an evaluation of EVS for reptiles of Guatemala.

We compared reptilian faunal composition between regions using Jaccard’s similarity index (Magurran 2004), which uses presence-absence data and is expressed as:

$C_{j}=\frac{a}{a+b+c}$

where *a* = the number of species shared between the two sites under comparison, *b* = number of species exclusive to the first site, and *c* = number of species exclusive to the second site. The index ranges from zero to one, with zero indicating that no species are shared between the sites being compared, and one indicating that all species are found in both sites. We plotted a dendrogram using PAST software version 2.17c (Hammer et al. 2001) to represent the relationship between sites in terms of their similarity in species composition according to the Jaccard index. For this analysis, we only included native species.

## Results

### Species richness, distribution, and conservation status

A total of 141 native reptile species belonging to 81 genera and 29 families has been recorded at the Selva Zoque region. These comprise 62 species of lizards, 70 snakes, seven turtles, and two crocodilians (Appendix 1). The best represented families are Dipsadidae and Colubridae, with 29 and 25 species, respectively, followed by Dactyloidae with 18 species, Phrynosomatidae with nine species, and Viperidae with seven species. The families with the fewest species in the region are Eublepharidae, Helodermatidae, Mabuyidae, Phyllodactylidae, Scincidae, Boidae, Natricidae, Sybinophiidae, Leptotyphlopidae, Loxocemidae, Dermatemydidae, Emydidae, and Geoemydidae, with one species each one. Three non-native species have been recorded in the region, the lizards *Anolissagrei* Duméril & Bibron *Gehyramutilata* (Wiegmann) and *Hemidactylusfrenatus* Duméril and Bibron. During our fieldwork (2013–2014), we recorded 48 species (Appendix 1), although all of them had been previously recorded.

Reptiles have been recorded mostly on the periphery of the study region, notably on western, southern and eastern end portions. In the northwest, northeast, and west, reptile collections are located below 1000 m a.s.l., while in the southeast, most of collections are between 1000 and 2000 m a.s.l. The central portion of Chimalapas, the mountainous zone known as Espinazo del Diablo in Uxpanapa and a zone between El Ocote Biosphere Reserve and UC zones corresponding to the northwest extreme of Cintalapa in Chiapas, remain with no collection of reptiles (Fig. 1). The distribution of eleven of the 141 native species (8%) is restricted to the Selva Zoque region, 26 species (18%) are distributed in the northern part of Mesoamerica that corresponds to Mexico, 66 species (47%) are distributed on Mesoamerica, and 38 species (27%) have a wide distribution, extending beyond Mesoamerica (Appendix 1).

Of the reptile species recorded in the Selva Zoque region, 60 species (42%) are in high-risk categories according to SEMARNAT: 39 species in the Subject to Special Protection category (Pr), 18 species are in the Threatened category (A) and three are Endangered (P). According to the IUCN Red List, six species (4%) are included in high-risk categories: three are Vulnerable (VU), two are Endangered (EN), and one is Critically Endangered (CR). Additionally, seven species are in the Data Deficient category (DD) and 23 species have not been evaluated (NE). The remaining 105 species are in low-risk categories. According to the EVS system, 28 species (20%) are in the high vulnerability category (Appendix 1).

### Comparison of the richness, composition, and conservation status of the reptile species from the Selva Zoque region with that of other tropical regions

With 141 native species recorded, the Selva Zoque region harbors the highest reptile species richness among the tropical regions considered in this study, surpassing Los Tuxtlas (113 species). The Mayan Forest ranks third (107 species), followed by La Lacandona (89 species), La Sepultura (79 species), Calakmul (73 species), Sian Ka´an (63 species), and finally Las Choapas (56 species; Table 1). The Los Tuxtlas Biosphere Reserve, with four species, has the highest diversity of non-native species, followed by Selva Zoque and Calakmul with three. La Sepultura, Sian Ka´an, and Las Choapas have two non-native species, and the Mayan Forest and Lacandona each have only one.

**Table 1.** Taxonomic composition of native reptile species recorded in the Selva Zoque region and seven other regions in northern Mesoamerica. Non-native species are not included in the taxonomic composition data.

Tropical region	Orders	Families	Genera	Lizards	Snakes	Turtles	Crocodilians	Native species	Non-native species
Selva Zoque	3	29	81	62	70	7	2	141	3
Los Tuxtlas	3	29	75	35	63	14	1	113	4
Mayan Forest	3	26	70	35	61	9	2	107	1
Lacandona	3	25	64	28	52	7	2	89	1
La Sepultura	2	25	59	29	47	3	0	79	2
Calakmul	3	21	51	24	39	9	1	73	3
Sian Ka´an	3	23	51	25	26	10	2	63	2
Las Choapas	3	20	41	20	28	7	1	56	2

**Figure 2.**
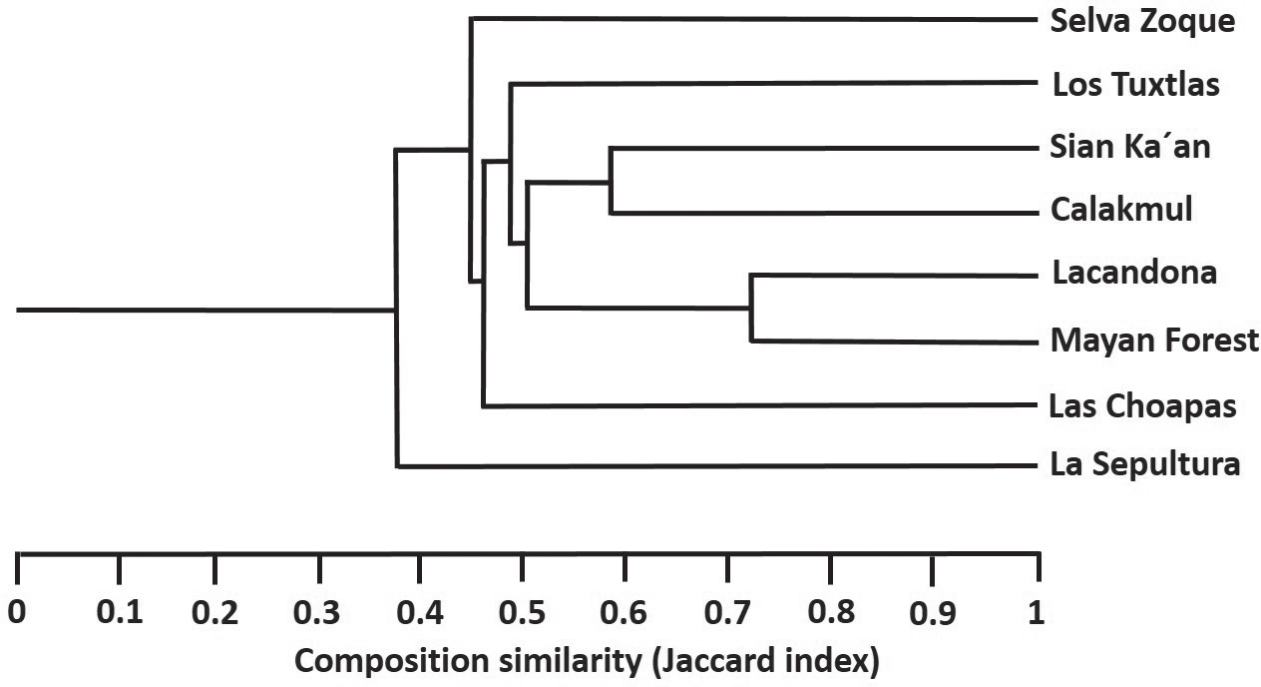
Similarity in reptile species composition for eight tropical areas in northern Mesoamerica.

The dendrogram indicates that La Sepultura has the most dissimilar species composition of the nine regions (Fig. 2). This Biosphere Reserve is followed by Selva Zoque, Las Choapas and Los Tuxtlas in terms of their dissimilarity in relation to the remaining regions. The four remaining regions in turn form a group with a value of Jaccard index [*Cj*] = 0.5 (Fig. 2). The composition analysis by pairs indicates that Los Tuxtlas had the species composition most similar to that of Selva Zoque, with 44% shared species (*Cj* = 0.44), followed by Lacandona (*Cj* = 0.39), whereas the region with the least similar species composition to Selva Zoque was Sian Ka´an (*Cj* = 0.20). The regions with the most similar species composition were Lacandona and Mayan Forest in Guatemala (*Cj* = 0.72), followed by Sian Ka´an and Calakmul (*Cj* = 0.58). The least similar regions were Sian Ka´an and La Sepultura (*Cj* = 0.14).

**Figure 3.**
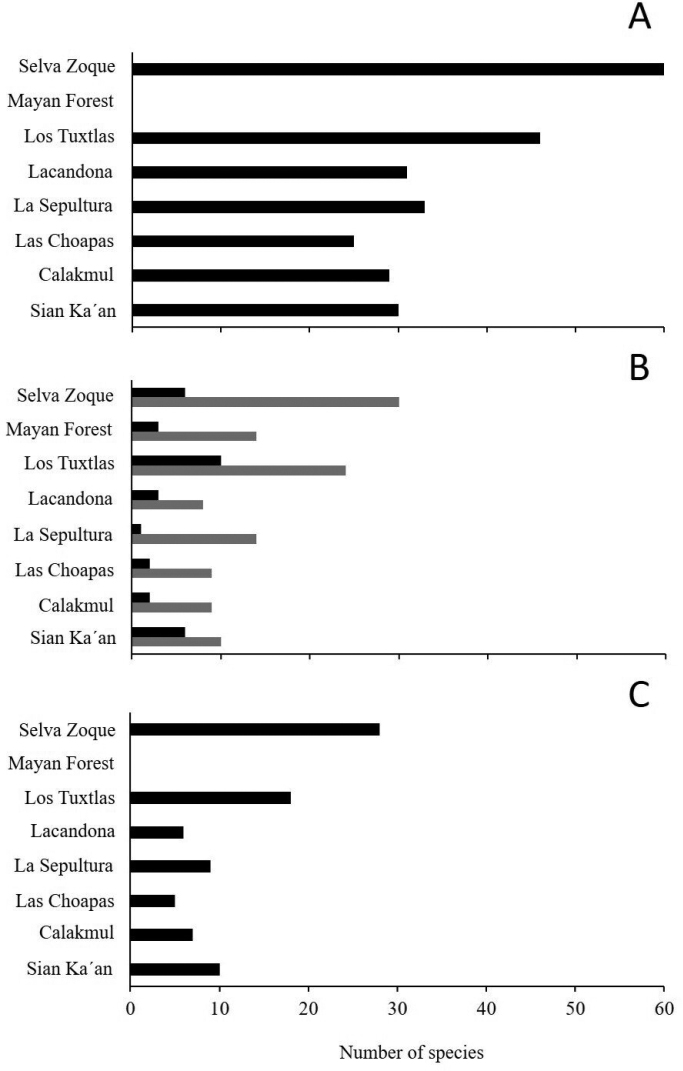
Number of reptile species in high extinction-risk categories according to **A** NOM-059-SEMARNAT-2010**B** IUCN Red List, and **C** the Environmental Vulnerability Score, for eight tropical regions in northern Mesoamerica. The black bars in the three plots represent the number of species in high extinction-risk categories, and the grey bars in the graph B represent the number of species not evaluated or in the Data Deficient category by the IUCN.

The Selva Zoque and Los Tuxtlas regions have the highest number of endemic species to the regions, with eleven each, followed by La Sepultura (three species) and Mayan Forest with one endemic species while the rest of regions do not have endemic species (Appendix 1). Also, the Selva Zoque region has the highest number of species in high-risk categories of extinction according to the NOM-059 criteria, with 60 species, followed by Los Tuxtlas with 46 species. The other regions have from 33 species (La Sepultura) to 25 species (Las Choapas) in high-risk categories (Fig. 3A). Based on the IUCN Red List, the number of reptiles in high-risk categories of extinction is highest in Los Tuxtlas, with ten species, and the other regions have from one to six species in these categories. The Selva Zoque region has the highest number of species classified as Not evaluated (NE) and with Data Deficient (DD) on the IUCN Red List with 30 species, followed by Los Tuxtlas (24 species); the remaining regions have from eight to 14 NE and DD species (Appendix 1; Fig. 3B). The Selva Zoque has the highest number of species (28) included in the high vulnerability category to environmental degradation, followed by Los Tuxtlas with 18 species; the remaining regions have between five and ten species (Fig. 3C).

## Discussion

Our results show that the Selva Zoque region harbors the greatest reptile species richness in northern Mesoamerica, with a distinctive species composition and several species inhabit exclusively on Selva Zoque region. Furthermore, one in every two reptile species that inhabit in Selva Zoque is threatened, is highly vulnerable or there is not sufficient information to know its extinction risk level. All this underscore the importance of the Selva Zoque region for reptile conservation in Mexico and Mesoamerica. This region also offers opportunities to study unexplored well-preserved tropical forest areas, as well as species for which little is known about their biology, ecology, and conservation status.

The Selva Zoque region is more diverse in native reptile species than even Los Tuxtlas, which has 28 fewer native species (López-Luna 2017). In the Selva Zoque region three exotic species (*Anolissagrei*, *Gehyramutilata*, *Hemidactylusfrenatus*) have been recorded, fewer than other regions such as Los Tuxtlas, which is located near the coast. The occurrence of the highest diversity of native species in some portions of Selva Zoque over that of other tropical regions has been previously observed in amphibians (Aguilar-López et al. 2016a). The 141 native reptile species recorded in the Selva Zoque region represent 16% of the 864 reptile species recorded in Mexico (Flores-Villela and García-Vázquez 2014), 52% of the 270 reptile species recorded in south-eastern Mexico (Johnson et al. 2010), and 11% of the 1284 species recorded in Mesoamerica (Johnson et al. 2018).

That said, the inventory of reptile species in the Selva Zoque region is far from complete, particularly in UC zones. In the last three years alone, three new species have been described (*Anolispurpuronectes* [Gray et al. 2016], *Chersodromusaustralis* [Canseco-Márquez et al. 2018] and *Cenaspisaenigma* [Campbell et al. 2018]), and more species await formal description. Furthermore, several zones within our study area lack reptile records entirely and remain unexplored, and hence additional surveys could reveal reptile species unrecorded for the region and perhaps altogether new species to science. As such, the species richness we report here is likely an underestimate, highlighting the need for additional sampling effort to complete the species inventory of the Selva Zoque region.

The high species richness in the Selva Zoque region may be the result of a series of factors. One is the notably complex orography with lowland zones, both on the Gulf of Mexico and on the Pacific versant, and also a series of mountain ranges of intermediate elevation–the Sierra Atravesada, the Espinazo del Diablo, the Sierra Tres Picos, and Cerro La Colmena (Wendt 1987; Ortíz-Pérez et al. 2004; SEMARNAT 2001)–with elevations from 100 to 2300 m a.s.l. There is a range of 11 °C in mean annual temperature across this elevation gradient, and a difference of 3600 mm in mean annual rainfall across the Selva Zoque (Beard 1955; SEMARNAT 2001). In addition, at least seven vegetation types exist in the region (Arriaga et al. 2000b; SEMARNAT 2001). Together, these factors create a wide variety of habitats for a large diversity of reptile species with different eco-physiological requirements and evolutionary histories.

Although the Selva Zoque did not result clustered with any of the regions in the similarity analysis, Los Tuxtlas and Lacandona Biosphere Reserves are the most similar tropical regions to the Selva Zoque. This pattern has been observed for amphibians in a comparison between Uxpanapa-Chimalapas zone and the same tropical regions (except from La Sepultura) used in this study, and can be explained by the high number of recorded species in these three sites and the high number of species that they share. The Selva Zoque reptile fauna has a combined component of species from two different biogeographic provinces (Morrone 2005): species found in the province of Gulf of Mexico (e.g., *Anolissericeus* Hallowell, *Holcosusamphigrammus* (Smith and Laufe), and those in the province of Mexican Pacific Coast (e.g., *Loxocemusbicolor* Cope, *Porthidiumdunni* (Hartweg and Oliver), *Rhinoclemmysrubida* (Cope)). The high percentage (8%) of endemism of reptile species to the Selva Zoque region may be due to its stable Pleistocene climate conditions, which allowed for the diversification of different biological groups (Lira-Torres et al. 2012; Rodríguez-Gómez et al. 2013). A pattern of high endemicity of the whole Selva Zoque region has been observed for mammals (Escalante 2003), but also in parts of Selva Zoque region like Uxpanapa-Chimalapas for other vertebrates as amphibians (Aguilar-López et al. 2016) or for Chimalapas zone in the case of birds (Peterson et al. 2003).

**Figure 4.**
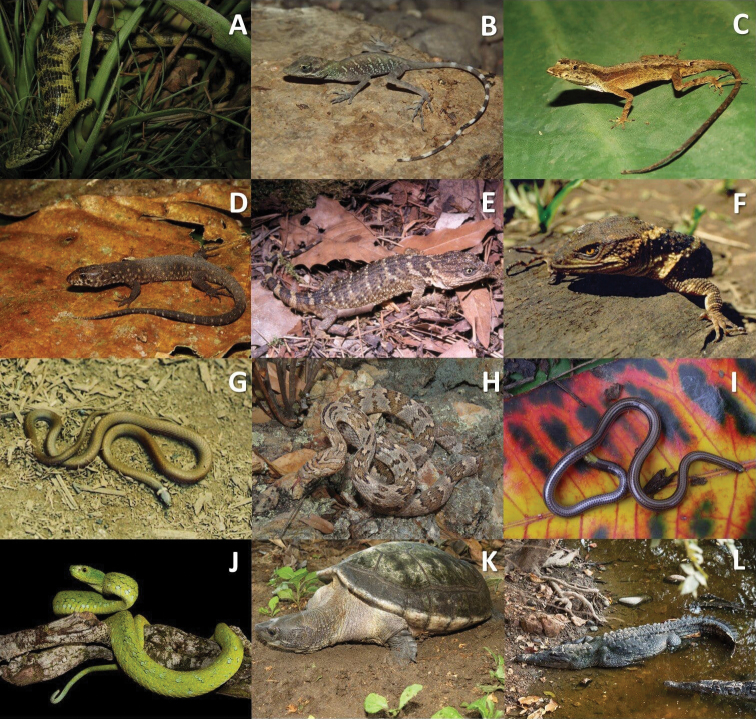
Reptile species in high extinction-risk categories by IUCN, NOM or EVS initiatives, or listed as Data Deficient or Not evaluated by the IUCN red list (see text) **A***Abroniabogerti***B***Anolisalvarezdeltoroi***C***Anolispygmaeus***D***Lepidophymatuxtlae***E***Xenosaurusarboreus***F***Xenosaurusrackhami***G***Tantillabriggsi***H***Trimorphodonbiscutatus***I***Epictiaphenops***J***Bothriechisrowleyi***K***Dermatemysmawii* and **L***Crocodylusacutus*.

Our results suggest that Selva Zoque is a priority conservation area for the reptile fauna of Mexico because a relatively high proportion (43%) of the species that inhabit there are in high-risk of extinction categories in the NOM-059; equivalent to 33% of all the Mexican reptile species included in this initiative (SEMARNAT 2010). Additionally, a moderate number of reptile species are in the high vulnerability category of environmental degradation (20%). In contrast, Selva Zoque does not harbor a high number of species in high-risk categories of extinction according to the IUCN. The differences in the number of species in high risk of extinction among initiatives is presumably because the NOM-059 does not consider the entire distribution of the species, it only takes into account the distribution of the species within the Mexican territory, which may lead to a restricted distribution, but only within Mexico, this does not necessarily reflect the entire distribution of the species. Additionally, populations of some reptile species that occur within Mexico may be scarce or may be declining, while populations of those species but outside of Mexico may be stable. Even so, the importance of Selva Zoque region lies in the relatively high proportion (21%) of species that are classified as Not evaluated (NE) and Data Deficient (DD). Because of the conservation status of vegetation in some areas of the region, it represents an opportunity for gathering information on the biology of these species, and this could contribute to their being assigned a category. This is the case for *Abroniabogerti* Tihen, *Anolisalvarezdeltoroi* Nieto Montes de Oca, *Xenosaurusarboreus* (Lynch and Smith), and *Tantillabriggsi* Savitsky and Smith (Fig. 4A, B, E, G), endemic species to the Selva Zoque region, or species with distribution in Mesoamerica as *Trimorphodonbiscutatus* (Duméril, Bibron and Duméril), and *Epictiaphenops* (Cope) (Fig. 4H, I). In any case, though widely distributed, some reptile species are considered threatened under three classification systems, such as *Anolispygmaeus* Alvarez del Toro & Smith *Bothriechisrowleyi* (Bogert), *Dermatemysmawii* Gray and *Crocodylusacutus* (Cuvier) (Fig. 4C, J, K, L), all of which have been reported for the other regions (Appendix 1; Fig. 3).

The extensive areas of well-preserved forest in Selva Zoque are mostly located in the Chimalapas zone and El Ocote Biosphere Reserve (Flamenco-Sandoval et al. 2007; Lira-Torres et al. 2012), and offer an opportunity not only for the conservation of reptile diversity, but also for all the biotic diversity that inhabits the Selva Zoque region. Conservation is under the aegis of community initiatives in Chimalapas and has been successful in recent decades (Monterrubio-Solís and Newing 2013); however, it is necessary to implement and subsequently strengthen conservation efforts in areas without any protection. For Uxpanapa and the intermediate zones between Uxpanapa and El Ocote Biosphere Reserve, the constant and accelerated transformation of the original vegetation cover into crops and pastureland (Flamenco-Sandoval et al. 2007; Hernández et al. 2013) underscores the urgent need to stop the advance of the agricultural frontier (Arriaga et al. 2000b).

The implementation of activities proven to be effective in the conservation of reptiles in transformed landscapes in the study region is desirable. These could include: 1) ecological restoration (Smith et al. 2015) to facilitate the recovery of the reptile communities over time, 2) the protection of vegetation remnants (Pulsford et al. 2017) that can harbor important components of overall species diversity, and 3) protection of buffer zones around water bodies (Semlitsch and Bodie 2003) that maintain populations of several species, and also constitute biological corridors (Burbrink et al. 1999). Finally, the protection of Uxpanapa portion of Selva Zoque can contribute to the connection of natural protected areas of different governance types in the western (community protected areas of the Chimalapas, in Oaxaca) and eastern (federal protected area El Ocote, in Chiapas) of Selva Zoque (García-Bañuelos et al. 2019). This idea fits completely with the objective of the Mesoamerican Biological Corridor, an international initiative that aims to maintain biological diversity, decrease habitat fragmentation, improve the connectivity of the landscapes and of the ecosystems in Mesoamerica and to impulse social and economic development (Miller et al. 2001).

## Appendix 1

**Table:** List of native reptile species recorded in the Selva Zoque region and seven other regions in northern Mesoamerica, their distribution range and conservation status. Abbreviations: a = species recorded during our fieldwork. LS = La Sepultura, LT = Los Tuxtlas, MF = Mayan Forest, SZ = Selva Zoque. NA = Species not considered in the EVS system. Exotic species recorded in the evaluated regions: *Anoliscristatellus* (Calakmul), *Anolissagrei* (Selva Zoque, Los Tuxtlas, Calakmul, Sian Ka´an and Mayan Forest), *Gehyramutilata* (Selva Zoque), *Hemidactylusfrenatus* (Selva Zoque, Las Choapas, Lacandona, Los Tuxtlas, Calakmul and Sian Ka´an), *Hemidactylusturcicus* (Los Tuxtlas, La Sepultura) and *Indotyphlopsbraminus* (Las Choapas, Los Tuxtlas).

Species	Distribution range	Selva Zoque	Las Choapas	Lacandona	Los Tuxtlas	La Sepultura	Calakmul	Sian Ka'an	Mayan Forest	NOM-059	IUCN RED LIST	EVS	Category of vulnerability according to the EVS
**CLASS REPTILIA**													
**Order Squamata**													
**Suborder Lacertilia**													
** Anguidae **													
*Abroniabogerti* Tihen	SZ	1	0	0	0	0	0	0	0	P	Data Deficient	18	High
*Abroniachiszari* Smith & Smith	LT	0	0	0	1	0	0	0	0	P	Endangered	17	High
*Abroniamorenica* Clause, Luna-Reyes & Nieto-Montes de Oca	LS	0	0	0	0	1	0	0	0		Not evaluated	NA	NA
*Abroniaornelasi* Campbell	SZ	1	0	0	0	0	0	0	0	P	Data Deficient	18	High
*Abroniaramirezi* Campbell	LS	0	0	0	0	1	0	0	0		Data Deficient	18	High
*Abroniareidi* Werler & Shannon	LT	0	0	0	1	0	0	0	0	P	Data Deficient	18	High
*Celestusenneagrammus* (Cope)	MAMx	1	0	0	0	0	0	0	0	Pr	Least Concern	14	High
*Celestusingridae* (Werler & Campbell)	LT	0	0	0	1	0	0	0	0		Data Deficient	17	High
*Celestusrozellae* (Smith) ^a^	MA	1	0	1	0	0	0	0	1	Pr	Least Concern	13	Medium
*Gerrhonotusliocephalus* Wiegmann	MAMx	1	0	0	1	1	0	0	0	Pr	Least Concern	6	Low
** Corytophanidae **													
*Basiliscusvittatus* Wiegmann ^a^	WD	1	1	1	1	1	1	1	1		Least Concern	7	Low
*Corytophanescristatus* (Merrem)	WD	0	0	1	0	0	1	1	1	Pr	Least Concern	11	Medium
*Corytophaneshernandesii* (Wiegmann) ^a^	MA	1	1	1	1	1	1	0	1	Pr	Least Concern	13	Medium
*Laemanctuslongipes* Wiegmann	MA	1	0	1	1	0	1	0	1	Pr	Least Concern	9	Low
*Laemanctusserratus* Cope	MA	0	0	0	1	0	1	1	0	Pr	Least Concern	8	Low
** Dactyloidae **													
*Anolisalvarezdeltoroi* Nieto-Montes de Oca ^a^	MAMx	1	0	0	0	0	0	0	0		Data Deficient	17	High
*Anolisbarkeri* (Schmidt)	MAMx	1	1	0	1	0	0	0	0	Pr	Vulnerable	15	High
*Anolisbeckeri* Boulenger ^a^	MA	1	1	1	1	0	1	0	1		Least Concern	12	Medium
*Anolisbiporcatus* (Wiegmann) ^a^	WD	1	1	1	1	0	1	1	1	Pr	Not evaluated	10	Medium
*Anolisboulengerianus* Thominot	MAMx	1	0	0	0	0	0	0	0	Pr	Data Deficient	16	High
*Anoliscapito* Peters	MA	1	0	1	0	0	0	0	1		Least Concern	13	Medium
*Anoliscompressicaudus* Smith & Kerster ^a^	MAMx	1	0	0	0	0	0	0	0		Least Concern	15	High
*Anoliscuprinus* Smith	MAMx	1	0	0	0	1	0	0	0	Pr	Least Concern	16	High
*Anolisduellmani* Fitch & Henderson	LT	0	0	0	1	0	0	0	0	Pr	Data Deficient	17	High
*Anolislaeviventris* (Wiegmann)	MA	1	0	0	1	1	0	0	1		Not evaluated	9	Low
*Anolislemurinus* Cope ^a^	MA	1	1	1	1	0	1	1	1		Least Concern	8	Low
*Anolismatudai* Smith	MA	0	0	0	0	1	0	0	0	A	Least Concern	13	Medium
*Anolisparvicirculatus* Alvarez del Toro & Smith	SZ	1	0	0	0	0	0	0	0	A	Least Concern	16	High
*Anolispetersii* Bocourt	MA	1	0	0	1	0	0	0	0		Not evaluated	9	Low
*Anolispurpuronectes* Gray, Meza-Lázaro, Poe & Nieto-Montes de Oca ^a^	SZ	1	0	0	0	0	0	0	0		Not evaluated	16	High
*Anolispygmaeus* Alvarez del Toro & Smith ^a^	MAMx	1	0	0	0	0	0	0	0	Pr	Endangered	16	High
*Anolisrodriguezii* Bocourt ^a^	MA	1	1	1	1	0	1	1	1		Least Concern	10	Medium
*Anolissericeus* Hallowell ^a^	MA	1	1	0	1	1	1	1	0		Least Concern	8	Low
*Anolisspilorhipis* (Alvarez del Toro & Smith)	MAMx	1	0	0	0	0	0	0	0		Not evaluated	NA	NA
*Anolistropidonotus* Peters	MA	0	1	1	1	0	1	1	1		Least Concern	7	Low
*Anolisuniformis* Cope	MA	0	0	1	1	0	0	0	1		Least Concern	13	Medium
*Anolisunilobatus* Köhler & Vesely	MA	1	0	1	0	0	0	0	1		Least Concern	7	Low
** Eublepharidae **													
*Coleonyxelegans* Gray ^a^	MA	1	0	1	1	1	1	1	1	A	Least Concern	9	Low
** Gymnophthalmidae **													
*Gymnophthalmusspeciosus* (Hallowell)	WD	0	0	0	0	1	0	0	0	Pr	Least Concern	9	Low
** Helodermatidae **													
*Helodermahorridum* (Wiegmann)	MA	1	0	0	0	1	0	0	0	A	Least Concern	11	Medium
** Iguanidae **													
*Cachryxdefensor* (Cope)	MA	0	0	0	0	0	1	1	0	P	Vulnerable	15	High
*Ctenosauraacanthura* (Shaw)	MA	1	1	0	1	0	0	0	0	Pr	Least Concern	12	Medium
*Ctenosaurapectinata* (Wiegmann)	MA	1	0	0	0	0	0	0	0	A	Least Concern	15	High
*Ctenosaurasimilis* (Gray)	WD	1	0	1	0	1	0	1	1	A	Least Concern	8	Low
*Iguanaiguana* (Linnaeus) ^a^	WD	1	1	1	1	1	0	0	1	Pr	Least Concern	12	Medium
** Mabuyidae **													
*Marisoralineola* McCranie, Matthews & Hedges	MA	0	1	1	1	0	1	1	1		Not evaluated	NA	NA
*Marisorasyntoma* McCranie, Matthews & Hedges	MAMx	1	0	0	0	1	0	0	0		Not evaluated	NA	NA
** Phyllodactylidae **													
*Phyllodactylusmagnus* Taylor	MAMx	1	0	0	0	1	0	1	0		Not evaluated	NA	NA
*Thecadactylusrapicauda* (Houttuyn)	WD	0	0	1	0	0	1	1	1	Pr	Least Concern	10	Medium
** Phrynosomatidae **													
*Phrynosomaasio* Cope	MA	1	0	0	0	0	0	0	0	Pr	Least Concern	11	Medium
*Sceloporuscarinatus* Smith	MA	1	0	0	0	1	0	0	0		Least Concern	12	Medium
*Sceloporuschrysostictus* Cope	MA	0	0	0	0	0	1	1	1		Least Concern	13	Medium
*Sceloporuscozumelae* Jones	MAMx	0	0	0	0	0	0	1	0	Pr	Least Concern	15	High
*Sceloporusinternasalis* Smith & Bumzahem ^a^	MA	1	0	0	0	0	0	0	0		Least Concern	11	Medium
*Sceloporuslundelli* Smith	MA	0	0	0	0	0	1	1	1		Least Concern	14	High
*Sceloporusmelanorhinus* Bocourt	MA	1	0	0	0	1	0	0	0		Least Concern	9	Low
*Sceloporussalvini* Günther	MAMx	0	0	0	1	0	0	0	0	A	Data Deficient	15	High
*Sceloporusserrifer* Cope	WD	0	0	1	0	1	0	0	1	A	Least Concern	6	Low
*Sceloporussiniferus* Cope	MAMx	1	0	0	0	1	0	0	0		Least Concern	11	Medium
*Sceloporussmithi* Hartweg & Oliver	MAMx	1	0	0	0	0	0	0	0		Least Concern	15	High
*Sceloporusteapensis* Günther	MA	1	0	1	1	0	0	0	1		Least Concern	13	Medium
*Sceloporusvariabilis* Wiegmann ^a^	WD	1	1	0	0	1	0	0	0		Least Concern	5	Low
*Urosaurusbicarinatus* (Duméril)	MA	1	0	0	0	1	0	0	0		Least Concern	12	Medium
** Scincidae **													
*Mesoscincusschwartzei* (Fischer)	MAMx	0	0	1	0	0	1	1	1		Least Concern	11	Medium
*Plestiodonsumichrasti* (Cope) ^a^	MA	1	0	1	1	0	1	1	1		Least Concern	12	Medium
*Scincellaassata* (Cope)	MA	1	0	0	0	1	0	0	0		Least Concern	7	Low
*Scincellacherriei* (Cope) ^a^	MA	1	1	1	1	0	1	1	1		Least Concern	8	Low
*Scincellagemmingeri* (Cope)	MAMx	1	0	0	1	0	0	0	0		Least Concern	11	Medium
*Scincellaincerta* (Stuart)	MA	0	0	0	0	1	0	0	0		Least Concern	13	Medium
*Scincellasilvicola* (Taylor)	MAMx	1	0	0	0	0	0	0	0	A	Least Concern	12	Medium
** Sphaerodactylidae **													
*Gonatodesalbogularis* (Duméril & Bibron)	WD	1	0	0	0	0	0	0	0	Pr	Least Concern	11	Medium
*Sphaerodactylusglaucus* Cope	MA	1	1	1	1	1	1	1	1	Pr	Least Concern	12	Medium
*Sphaerodactylusmillepunctatus* (Hallowell)	MA	0	0	1	0	0	0	0	1		Least Concern	10	Medium
** Teiidae **													
*Aspidoscelisangusticeps* (Cope)	MA	0	0	0	0	0	1	1	1		Least Concern	13	Medium
*Aspidoscelisdeppei* (Wiegmann)	MA	1	1	0	1	1	1	0	0		Least Concern	8	Low
*Aspidoscelisguttata* (Wiegmann)	MAMx	1	0	0	1	1	0	0	0		Least Concern	12	Medium
*Aspidoscelismaslini* (Fritts)	MA	0	0	0	0	0	0	1	1	A	Least Concern	15	High
*Aspidoscelismotaguae* (Sackett)	MA	1	0	0	0	0	0	0	0		Least Concern	12	Medium
*Holcosusamphigrammus* (Smith and Laufe) ^a^	MAMx	1	1	0	1	0	0	0	0		Not evaluated	11	Medium
*Holcosuschaitzami* (Stuart)	MA	0	0	0	0	0	0	0	1		Data Deficient	NA	NA
*Holcosusfestivus* (Lichtenstein & Martens)	WD	0	0	1	0	0	0	0	1		Least Concern	11	Medium
*Holcosusgaigeae* (Smith & Laufe)	MAMx	0	0	0	0	0	1	1	0		Not evaluated	13	Medium
*Holcosushartwegi* (Smith)	MA	0	0	1	0	0	0	0	1		Not evaluated	12	Medium
*Holcosusparvus* (Barbour & Noble)	MA	1	0	0	0	1	0	0	0		Not evaluated	13	Medium
** Xantusiidae **													
*Lepidophymaflavimaculatum* Duméril	MA	1	1	1	0	0	0	1	1	Pr	Least Concern	8	Low
*Lepidophymalipetzi* Smith & Alavarez del Toro	SZ	1	0	0	0	0	0	0	0	A	Endangered	16	High
*Lepidophymamayae* Bezy	MA	0	0	0	0	0	0	0	1		Near Threatened	NA	NA
*Lepidophymapajapanensis* Werler ^a^	MAMx	1	1	0	1	0	0	0	0	Pr	Least Concern	13	Medium
*Lepidophymasmithii* Bocourt	MA	1	0	0	0	1	0	0	0	Pr	Least Concern	8	Low
*Lepidophymatuxtlae* Werler & Shannon ^a^	MAMx	1	1	0	1	0	0	0	0	A	Data Deficient	11	Medium
** Xenosauridae **													
*Xenosaurusarboreus* (Lynch & Smith)	SZ	1	0	0	0	0	0	0	0		Not evaluated	17	High
*Xenosaurusrackhami* Stuart	MA	1	0	0	0	0	0	0	0		Not evaluated	11	Medium
*Xenosaurussanmartinensis* Werler & Shannon	LT	0	0	0	1	0	0	0	0		Not evaluated	17	High
**Suborder Serpentes**													
** Boidae **													
*Boaimperator* Daudin	WD	1	1	1	1	1	1	1	1		Least Concern	10	Medium
** Dipsadidae **													
*Adelphicoslatifasciatum* Lynch & Smith	LS	0	0	0	0	1	0	0	0	Pr	Data Deficient	15	High
*Adelphicosquadrivirgatum* Jan	MA	1	0	1	0	1	0	0	1		Least Concern	10	Medium
*Adelphicosvisoninum* (Cope)	MA	0	1	0	1	0	0	0	0		Least Concern	12	Medium
*Amastridiumsapperi* (Werner) ^a^	MA	1	0	1	1	0	0	0	1		Least Concern	10	Medium
*Cenaspisaenigma* Campbell, Smith & Hall	SZ	1	0	0	0	0	0	0	0		Not evaluated	16	High
*Chersodromusaustralis* Canseco-Márquez, Ramírez-González & Campbell	SZ	1	0	0	0	0	0	0	0		Not evaluated	16	High
*Cleliaclelia* (Daudin)	WD	0	0	0	0	0	0	0	1		Least Concern	8	Low
*Cleliascytalina* (Cope) ^a^	MA	1	1	1	1	1	0	0	0		Least Concern	13	Medium
*Coniophanesbipunctatus* (Günther)	MA	0	1	1	1	1	0	0	1		Least Concern	10	Medium
*Coniophanesfissidens* (Günther) ^a^	WD	1	1	1	1	1	0	0	1		Least Concern	7	Low
*Coniophanesimperialis* (Baird & Girard) ^a^	WD	1	1	1	1	0	1	1	1		Least Concern	8	Low
*Coniophanespiceivittis* Cope	MA	1	0	0	1	1	0	0	0		Least Concern	7	Low
*Coniophanesquinquevittatus* (Duméril, Bibron & Duméril)	MA	0	0	1	1	0	0	0	1		Least Concern	13	Medium
*Coniophanesschmidti* Bailey	MA	0	0	1	0	0	1	1	1		Least Concern	13	Medium
*Conophislineatus* (Duméril, Bibron & Duméril)	MA	0	0	0	1	0	0	0	1		Least Concern	9	Low
*Conophis morai7* Pérez-Higareda, López-Luna & Smith	LT	0	0	0	1	0	0	0	0		Data Deficient	17	High
*Conophisvittatus* Peters	MA	0	0	0	0	1	0	0	0		Least Concern	11	Medium
*Dipsasbrevifacies* (Cope)	MA	0	0	0	0	0	1	1	0	Pr	Least Concern	15	High
*Enuliusflavitorques* (Cope)	WD	0	0	0	0	1	0	0	0		Least Concern	5	Low
*Geophiscarinosus* Stuart	MA	1	0	1	1	0	0	0	0		Least Concern	8	Low
*Geophisjuliai* Pérez-Higareda, Smith & López-Luna	LT	0	0	0	1	0	0	0	0		Vulnerable	13	Medium
*Geophislaticinctus* Smith & Williams	MAMx	1	0	0	0	0	0	0	0	Pr	Least Concern	11	Medium
*Geophis* sp. nov. ^a^	SZ	1	0	0	0	0	0	0	0		Not evaluated	16	High
*Imantodescenchoa* (Linnaeus) ^a^	WD	1	1	1	1	0	1	1	1	Pr	Least Concern	6	Low
*Imantodesgemmistratus* (Cope)	WD	1	0	1	1	1	1	0	1	Pr	Least Concern	6	Low
*Imantodestenuissimus* (Cope)	MAMx	0	0	0	0	0	1	0	0	Pr	Least Concern	13	Medium
*Leptodeiraannulata* (Linnaeus)	WD	1	1	0	1	1	0	0	0	Pr	Least Concern	6	Low
*Leptodeirafrenata* (Cope)	MA	0	1	1	1	0	1	1	1		Least Concern	12	Medium
*Leptodeiramaculata* (Hallowell)	MA	0	0	0	0	1	0	0	0	Pr	Least Concern	7	Low
*Leptodeiranigrofasciata* Günther	MA	1	0	0	0	0	0	0	0		Least Concern	8	Low
*Leptodeirapolysticta* (Günther) ^a^	MA	1	1	0	1	0	0	0	1		Not evaluated	6	Low
*Leptodeiraseptentrionalis* (Kennicott)	WD	0	0	1	0	1	1	0	0		Least Concern	8	Low
*Manolepisputnami* (Jan)	MAMx	1	0	0	0	1	0	0	0		Least Concern	13	Medium
*Niniadiademata* Baird & Girard ^a^	MA	1	1	1	1	0	1	0	1		Least Concern	9	Low
*Niniasebae* (Duméril, Bibron & Duméril) ^a^	MA	1	1	1	1	0	1	1	1		Least Concern	5	Low
*Oxyrhopuspetolarius* (Linnaeus) ^a^	WD	1	1	1	1	0	0	0	1		Least Concern	14	High
*Pliocercuselapoides* Cope	WD	1	0	1	1	1	1	1	1		Least Concern	10	Medium
*Rhadinaeadecorata* (Günther) ^a^	WD	1	1	1	1	0	0	0	1		Least Concern	9	Low
*Rhadinaeamacdougalli* Smith & Langebartel	SZ	1	0	0	0	0	0	0	0	Pr	Data Deficient	12	Medium
*Rhadinellaanachoreta* (Smith & Campbell)	MA	0	0	0	0	0	0	0	1		Least Concern	NA	NA
*Rhadinellagodmani* (Günther)	MA	1	0	0	0	0	0	0	0		Least Concern	10	Medium
*Sibondimidiatus* (Günther) ^a^	MA	1	0	1	1	0	0	0	1		Least Concern	10	Medium
*Sibonlinearis* Pérez-Higareda, López-Luna & Smith	LT	0	0	0	1	0	0	0	0		Data Deficient	16	High
*Sibonnebulatus* (Linnaeus)	WD	1	0	1	1	0	1	0	1		Least Concern	5	Low
*Sibonsanniolus* (Cope)	MA	0	0	0	0	0	1	1	1		Least Concern	12	Medium
*Tretanorhinusnigroluteus* Cope	MA	0	0	1	1	0	0	1	1		Least Concern	10	Medium
*Tropidodipsasfasciata* Günther	WD	1	0	0	1	1	1	0	0		Least Concern	13	Medium
*Tropidodipsasfischeri* (Boulenger)	MA	0	0	0	0	0	0	0	0		Least Concern	11	Medium
*Tropidodipsassartorii* Cope	MA	1	1	1	1	0	1	1	1	Pr	Least Concern	9	Low
*Xenodonrabdocephalus* (Wied-Neuwied)	WD	1	1	1	1	0	1	0	1		Least Concern	13	Medium
** Colubridae **													
*Coluberconstrictor* Linnaeus	WD	1	0	0	0	0	0	0	1	A	Least Concern	10	Medium
*Dendrophidionrufiterminorum* Cadle & Savage	MA	0	0	0	0	0	0	0	1		Not evaluated	NA	NA
*Dendrophidionvinitor* Smith ^a^	MA	1	0	0	1	0	0	0	1		Least Concern	13	Medium
*Drymarchonmelanurus* (Duméril, Bibron & Duméril) ^a^	WD	1	1	1	1	1	1	1	1		Least Concern	6	Low
*Drymobiuschloroticus* (Cope)	MA	1	0	0	1	1	0	0	0		Least Concern	8	Low
*Drymobiusmargaritiferus* (Schlegel)	WD	1	1	1	1	1	1	1	1		Least Concern	6	Low
*Ficimiapublia* Cope	MA	1	1	1	1	0	1	1	1		Least Concern	9	Low
*Ficimiavariegata* (Günther)	MAMx	0	0	0	1	0	0	0	0		Data Deficient	14	High
*Masticophismentovarius* (Duméril, Bibron & Duméril)	WD	1	1	1	1	1	0	0	1	A	Least Concern	6	Low
*Mastigodryasmelanolomus* (Cope) a	WD	1	1	1	1	1	1	1	1		Least Concern	6	Low
*Lampropeltisabnorma* (Bocourt)	MA	1	1	1	1	1	1	0	1		Least Concern	9	Low
*Leptophisahaetulla* (Linnaeus) ^a^	WD	1	0	1	1	0	1	1	1	A	Least Concern	10	Medium
*Leptophisdiplotropis* (Günther)	MAMx	0	0	0	0	1	0	0	0	A	Least Concern	14	High
*Leptophismexicanus* Duméril, Bibron & Duméril	WD	1	1	1	1	1	1	1	1	A	Least Concern	6	Low
*Oxybelispotosiensis* (Taylor)	MAMx	1	0	1	1	1	1	1	1		Not evaluated	NA	NA
*Oxybelisfulgidus* (Daudin)	WD	1	0	1	1	1	1	0	1		Least Concern	9	Low
*Phrynonaxpoecilonotus* (Günther)	MA	1	0	1	1	0	1	1	1		Least Concern	10	Medium
*Pituophislineaticollis* (Cope)	MA	1	0	0	0	1	0	0	0		Least Concern	8	Low
*Pseudelapheflavirufa* (Cope)	MA	1	0	1	1	1	1	0	1		Least Concern	10	Medium
*Salvadoralemniscata* (Cope)	MA	1	0	0	0	1	0	0	0	Pr	Least Concern	15	High
*Senticolistriaspis* (Cope)	WD	1	0	1	1	1	1	0	1		Least Concern	6	Low
*Spilotespullatus* (Linnaeus) ^a^	WD	1	0	1	1	1	1	1	1		Least Concern	6	Low
*Stenorrhinadegenhardtii* (Berthold) ^a^	WD	1	0	1	1	0	0	0	1		Least Concern	9	Low
*Stenorrhinafreminvillei* (Duméril, Bibron & Duméril)	MA	1	0	1	0	1	0	0	1		Least Concern	7	Low
*Symphimusleucostomum* Cope	MAMx	0	0	0	0	1	0	0	0	Pr	Least Concern	14	High
*Symphimusmayae* (Gaige)	MA	0	0	0	0	0	1	1	0	Pr	Least Concern	14	High
*Tantillabriggsi* Savitzky & Smith	SZ	1	0	0	0	0	0	0	0	A	Data Deficient	16	High
*Tantillacuniculator* Smith	MA	0	0	0	0	0	0	0	1	Pr	Least Concern	NA	NA
*Tantillajani* (Günther)	MA	0	0	0	0	1	0	0	0		Vulnerable	12	Medium
*Tantillamoesta* (Günther)	MA	0	0	0	0	0	0	0	1		Least Concern	NA	NA
*Tantillarubra* Cope	MA	1	0	0	0	1	0	0	0	Pr	Least Concern	5	Low
*Tantillaschistosa* (Bocourt)	MA	0	0	1	1	0	0	0	1		Least Concern	8	Low
*Tantillaslavensi* Pérez-Higareda, Smith & Smith	LT	0	0	0	1	0	0	0	0	Pr	Data Deficient	14	High
*Tantillatecta* Campbell & Smith	MF	0	0	0	0	0	0	0	1		Data Deficient	NA	NA
*Tantillitabrevissima* (Taylor)	MA	0	0	0	0	1	0	0	0	Pr	Least Concern	9	Low
*Tantillitacanula* (Cope)	MA	0	0	0	0	0	1	0	1		Least Concern	12	Medium
*Tantillitalintoni* (Smith) ^a^	MA	1	0	1	1	0	1	0	1	Pr	Least Concern	12	Medium
*Trimorphodonbiscutatus* (Duméril, Bibron & Duméril)	MA	1	0	0	1	1	0	0	0	A	Not evaluated	7	Low
** Natricidae **													
*Nerodiarhombifer* (Hallowell)	WD	0	0	1	1	0	0	0	0		Least Concern	10	Medium
*Thamnophiscyrtopsis* (Kennicott)	WD	1	0	0	0	0	0	0	0	A	Least Concern	7	Low
*Thamnophismarcianus* (Baird & Girard)	WD	0	0	1	0	0	1	0	0	A	Least Concern	10	Medium
*Thamnophisproximus* (Say)	WD	0	1	0	1	1	0	1	1	A	Least Concern	7	Low
**Sybinophiidae**													
*Scaphiodontophisannulatus* (Duméril, Bibron & Duméril) ^a^	WD	1	0	1	1	1	0	0	1		Least Concern	11	Medium
** Elapidae **													
*Micrurusapiatus* (Jan)	MAMx	0	0	1	0	0	1	1	1		Not evaluated	NA	NA
*Micrurusbrowni* Schmidt & Smith	MA	1	0	0	0	1	0	0	0	Pr	Least Concern	8	Low
*Micrurusdiastema* (Duméril, Bibron & Duméril) ^a^	MA	1	1	0	1	0	0	0	0	Pr	Least Concern	8	Low
*Micruruselegans* Jan	MA	1	1	1	1	0	0	0	0	Pr	Least Concern	13	Medium
*Micruruslimbatus* Fraser	LT	0	0	0	1	0	0	0	0	Pr	Least Concern	17	High
** Leptotyphlopidae **													
*Epictiaphenops* (Cope)	MA	1	0	0	0	1	0	0	0		Not evaluated	4	Low
*Epictiaresetari* Wallach	MAMx	0	0	0	1	0	0	0	0		Not evaluated	11	Medium
** Loxocemidae **													
*Loxocemusbicolor* Cope	MA	1	0	0	0	1	0	0	0	Pr	Least Concern	10	Medium
** Typhlopidae **													
*Amerotyphlopsmicrostomus* (Cope)	MA	0	0	0	0	0	1	0	1		Least Concern	12	Medium
*Amerotyphlopstenuis* (Salvin)	MA	1	0	0	1	0	0	0	0		Least Concern	11	Medium
** Viperidae **													
*Agkistrodonbilineatus* Günther	MA	0	0	0	0	1	1	0	1	Pr	Near Threatened	11	Medium
*Bothriechisbicolor* (Bocourt)	MA	0	0	0	0	1	0	0	0	A	Least Concern	14	High
*Bothriechisrowleyi* (Bogert)	MAMx	1	0	0	0	0	0	0	0	Pr	Vulnerable	16	High
*Bothriechisschlegelii* (Berthold)	WD	1	0	1	0	0	0	0	1		Least Concern	12	Medium
*Bothropsasper* (Garman) ^a^	WD	1	1	1	1	1	1	1	1		Not evaluated	12	Medium
*Cerrophidiongodmani* (Günther)	MA	1	0	0	0	0	0	0	0		Least Concern	11	Medium
*Crotalusehecatl* Carbajal-Márquez, Cedeño-Vázquez, Martínez-Arce, Neri-Castro & Machkour-M'Rabet	MAMx	1	1	0	0	1	0	0	0		Not evaluated	NA	NA
*Crotalusmictlantecuhtli* Carbajal-Márquez, Cedeño-Vázquez, Martínez-Arce, Neri-Castro & Machkour-M'Rabet	LT	0	0	0	1	0	0	0	0		Not evaluated	NA	NA
*Crotalussimus* Latreille	MA	0	0	1	0	0	0	0	0		Least Concern	11	Medium
*Crotalustzabcan* Klauber	MAMx	0	0	0	0	0	1	1	1		Least Concern	NA	NA
*Metlapilcoatlusmexicanus* (Duméril, Bibron & Duméril)	MA	0	0	1	0	0	0	0	1		Least Concern	12	Medium
*Metlapilcoatlusolmec* (Pérez-Higareda, Smith & Julia-Zertuche) ^a^	MA	1	0	0	1	0	0	0	0	A	Least Concern	15	High
*Porthidiumdunni* (Hartweg & Oliver)	MAMx	1	0	0	0	1	0	0	0	A	Least Concern	16	High
*Porthidiumnasutum* (Bocourt)	WD	0	0	1	0	0	0	0	1	Pr	Least Concern	14	High
*Porthidiumyucatanicum* (Smith)	MA	0	0	0	0	0	0	1	0	Pr	Least Concern	17	High
**Order Testudines**													
**Suborder Cryptodira**													
** Cheloniidae **													
*Carettacaretta* (Linnaeus)	WD	0	0	0	1	0	0	1	0	P	Vulnerable	NA	NA
*Cheloniamydas* (Linnaeus)	WD	0	0	0	1	0	0	1	0	P	Endangered	NA	NA
*Eretmochelysimbricata* (Linnaeus)	WD	0	0	0	1	0	0	1	0	P	Critically Endangered	NA	NA
*Lepidochelyskempii* Garmin	WD	0	0	0	1	0	0	0	0	P	Critically Endangered	NA	NA
** Chelydridae **													
*Chelydrarossignonii* (Bocourt)	MA	0	1	1	1	0	0	0	1	Pr	Vulnerable	17	High
** Dermatemydidae **													
*Dermatemysmawii* Gray	MA	1	0	1	1	0	0	0	1	P	Critically Endangered	17	High
** Dermochelyidae **													
*Dermochelyscoriacea* (Vandelli)	WD	0	0	0	1	0	0	1	0	P	Vulnerable	NA	NA
** Emydidae **													
*Terrapenecarolina* (Linnaeus)	WD	0	0	0	0	0	1	0	0	Pr	Vulnerable	10	Medium
*Trachemysvenusta* (Gray)	WD	1	1	1	1	0	1	1	1	Pr	Not evaluated	13	Medium
** Geoemydidae **													
*Rhinoclemmysareolata* (Duméril, Bibron & Duméril)	MA	0	1	1	1	0	1	1	1	A	Near Threatened	13	Medium
*Rhinoclemmyspulcherrima* (Gray)	MA	0	0	0	0	1	0	0	0	A	Not evaluated	8	Low
*Rhinoclemmysrubida* (Cope)	MAMx	1	0	0	0	1	0	0	0	Pr	Near Threatened	14	High
** Kinosternidae **													
*Claudiusangustatus* Cope	MA	0	1	0	1	0	1	0	1	P	Near Threatened	14	High
*Kinosternonacutum* Gray ^a^	MA	1	1	1	1	0	1	0	1	Pr	Near Threatened	11	Medium
*Kinosternoncreaseri* Hartweg	MAMx	0	0	0	0	0	1	1	0		Least Concern	15	High
*Kinosternonleucostomum* (Duméril, Bibron & Duméril)	WD	1	1	1	1	0	1	1	1	Pr	Not evaluated	10	Medium
*Kinosternonscorpioides* (Linnaeus) ^a^	WD	1	0	0	1	1	1	1	1	Pr	Not evaluated	10	Medium
*Staurotypustriporcatus* (Wiegmann)	MA	1	1	1	1	0	1	1	1	A	Near Threatened	14	High
**Order Crocodylia**													
**Suborder Eusuchia**													
** Crocodylidae **													
*Crocodylusacutus* (Cuvier) ^a^	WD	1	0	1	0	0	0	1	1	Pr	Vulnerable	14	High
*Crocodylusmoreletii* (Duméril & Bibron)	WD	1	1	1	1	0	1	1	1	Pr	Least Concern	13	Medium
